# Treatment with Subcritical Water-Hydrolyzed Citrus Pectin Ameliorated Cyclophosphamide-Induced Immunosuppression and Modulated Gut Microbiota Composition in ICR Mice

**DOI:** 10.3390/molecules25061302

**Published:** 2020-03-12

**Authors:** Jianbing Chen, Chengcheng Zhang, Qile Xia, Daqun Liu, Xinghe Tan, Yingdi Li, Yan Cao

**Affiliations:** 1 College of Food Science and Technology, Hunan Agricultural University, Changsha 410128, China; chenjianb@zaas.ac.cn; 2 Key Laboratory of Post-Harvest Handling of Fruits, Ministry of Agriculture and Rural Affairs; Food Science Institute, Zhejiang Academy of Agricultural Sciences, Hangzhou 310021, China; zccwsf@126.com (C.Z.); cookxql@163.com (Q.X.); daqun.liu@hotmail.com (D.L.); 13589602278@126.com (Y.L.); caoyan_115@163.com (Y.C.)

**Keywords:** Subcritical-water, Citrus pectin, Cyclophosphamide, Immunosuppression, Gut microbiota

## Abstract

Subcritical water can effectively hydrolyze pectin into smaller molecules while still maintaining its functional regions. Pectic heteropolysaccharide can mediate immune regulation; however, the possible effects of subcritical water-hydrolyzed citrus pectin (SCP) on the immune response remain unclear. Therefore, the effects of SCP on immunomodulatory functions and intestinal microbial dysbiosis were investigated using a cyclophosphamide-induced immunosuppressed mouse model. In this research, immunosuppressed ICR mice were administrated with SCP at dosages of 300/600/1200 mg/kg.bw by oral gavage, and body weight, immune organ indexes, cytokines, and gut microbiota were determined. The results showed that subcritical water treatment decreased the molecular mass and increased the content of galacturonic acid in citrus pectin hydrolysates. Meanwhile, the treatment with SCP improved immunoregulatory functional properties and bioactivities over the original citrus pectin. For example, SCP protected immune organs (accelerated recovery of immune organ indexes) and significantly enhanced the expression of immune-related cytokines (IL-2, IL-6, IFN-γ, and TNF-α). The results of the 16S rDNA sequencing analysis on an IlluminaMiSeq platform showed that SCP normalized Cy-induced gut dysbiosis. SCP ameliorated Cy-dependent changes in the relative abundance of several taxa, shifting the balance back to normal status (e.g., SCP increased beneficial Muribaculaceae, Ruminococcaceae, Bacteroidaceae, and Prevotellaceae while decreasing pathogenic Brevundimonas and Streptococcus). The results of this study suggest an innovative application of citrus pectin as an immunomodulator.

## 1. Introduction

Immunosuppression is a state of temporary or permanent immune disorder, which can affect or destroy the immune functional balance, causing an immunocompromised state [1]. Immunosuppression is closely associated with upper respiratory tract infection, urinary tract infection, sepsis, and meningitis [2]. A recent study indicated that an imbalance of immune function was closely related to dysbiosis of the intestinal microbiota [3], as the gut barrier was disrupted and digestive system complications developed in the immunocompromised group [4]. The commensal microbiota plays an important role in the development and maintenance of the immune system and intestinal homeostasis via stimulating the immune response and maintaining the epithelial barrier functions [5]. Hence, gut microbiota dysbiosis is closely related to immunosuppression. For example, cyclophosphamide-induced immunosuppressed mice exhibited a decrease in the richness of bacterial species and an increase in the proportion of Bacteroidetes, while the proportion of Firmicutes and Proteobacteria increased [1,3,5]. This suggests that an imbalance of the immune system causes dysbiosis of the intestinal microbiota. Hence, optimizing the structure of gut microbiota may be an alternative target for ameliorating an imbalance in immunity.

Growing evidence indicates that polysaccharides from plants have immune regulating activity through the gut microbiota [6,7,8]. Polysaccharides are host-indigestible but can be metabolized by the gut microbiota. They are potential assets for the regulation of the gut microbiota as they promote the growth of beneficial bacteria (such as butyrate-producing bacteria) [5,9]. Citrus pectin is a complex polysaccharide that is widely used as a food additive. However, the application of citrus pectin in the medical field has been limited by its large molecular weight and poor absorption into the human digestive system [10]. Recent studies suggest that degradation or modification of pectin improved its functional properties and bioactivities compared to the native pectin [10,11]. For example, ultrasound pretreatment improved galactose content in citrus pectin hydrolysates, resulting in improved anti-cancer activity [12]. Additionally, the degradation product of hawthorn pectin enhanced the antioxidant activity of pectin [13]. Modified pectin contains smaller fragments with lower molecular mass and increased solubility, making it possible to enter the digestive system and exert its functions.

Conventional chemical, enzymatic, or physical methods are commonly used for pectin degradation [14]. Subcritical water treatment, an environmentally friendly technique, has been investigated as a method of pectin degradation in recent years. Subcritical water is liquid water at temperatures between the normal boiling point (100 °C) and the critical point (374 °C) [15]. Klinchongkon et al. [16] reported that subcritical water could effectively hydrolyze passion fruit pectin into smaller molecules, resulting in an increase of galacturonic acid content. Our previous study also found that subcritical water produced by high temperature and high-pressure sterilization, as is used in food processing, had a great influence on the macromolecular structure of citrus pectin, and the molecular weight of the citrus pectin decreased gradually. Hence, subcritical water can effectively hydrolyze pectin into smaller molecules while increasing the content of galacturonic acid in citrus pectin hydrolysates, making it possible to improve its functional properties and bioactivities. However, the possible effects of subcritical water-hydrolyzed citrus pectin (SCP) on the immune response remain unclear. Therefore, the objective of the present study was to evaluate the effects of SCP on facilitating immune function and its prebiotic effects on the gut microbiota in a cyclophosphamide (Cy)-induced immunosuppressed mouse model. The results of this study will provide an innovative method for the application of citrus pectin.

## 2. Results

### 2.1. Preliminary Characterization of SCP

**Figure 1:**
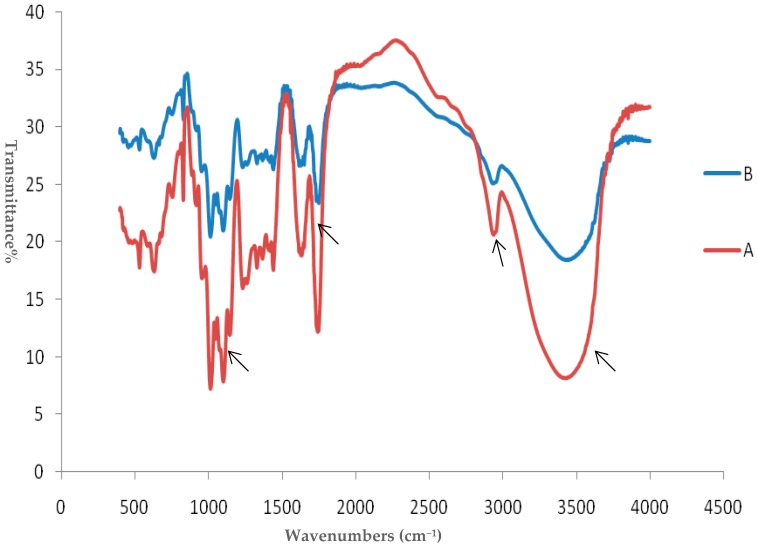
FT-IR spectrum of (**A**) subcritical water-hydrolyzed citrus pectin and (**B**) original citrus pectin.

The molecular mass of the original citrus pectin (CP) and the products of subcritical water treatment using a high-pressure pump was measured by size exclusion chromatography. The weight-average molecular weight (Mw) and number-average molecular weight (Mn) of citrus pectin were remarkably reduced (from 157 kDa to 123 kDa for Mw and 121 kDa to 86 kDa for Mn) within 40 min of heat treatment, indicating that subcritical water treatment resulted in a decrease in the molecular weight of pectin. Additionally, the galacturonic acid content of the citrus pectin samples increased from 64.8% to 69.9% (compared to the original CP) following subcritical water treatment. Figure 1 illustrates the infrared spectra of citrus pectin with and without subcritical water treatment. The broad absorption peak at 3432 cm^−1^ was contributed by the stretching vibration of the hydroxyl group [17]. Strong bands occurring at 343scm^−1^ represent the formation of O-H in the SCP sample. The methylesterified carbonyl groups (C–O) and the ionic carboxyl groups (COO–) occurred at 1735 cm^−1^, and 1636 cm^−1^, respectively [18]. Strong bands occurring at 1636 cm^−1^ and 1735 cm^−1^ represent a high content of carbonyl groups in the SCP samples and the esterification reaction with the hydroxyl group. As can be seen from Figure 1, the absorption peak at 2938 cm^−1^ due to –OCH3 was unchanged with subcritical water treatment, indicating that the DM of pectin did not change. Three absorption peaks at 1016 cm^−1^, 1104 cm^−1^ and 1148 cm^−1^indicate the pyranose configuration of pectin [19], and a high content of uronic acid in the SCP samples was found, as evidenced by the strong bands occurring at regions of 1000–1200 cm^−1^, which is in line with the galacturonic acid content analysis.

### 2.2. Effects of SCP on Body Weight and Immune Organ Indices

Table 1 shows the effects of SCP on the body weight and immune organ indices in cyclophosphamide-induced immunosuppressed mice. In the present study, immunosuppressed mice were treated with SCP at the dosages of low-dose (300 mg/kgbw, Cy + LSCP), middle-dose (600 mg/kg∙bw, Cy + MSCP), and high-dose (1200 mg/kgbw, Cy + HSCP) for 21 days; CP control group was treated with original CP (Cy + CP: 1200 mg/kg); the positive control group was injected subcutaneously with levamisole at a dose of 7 mg/kg bw (Cy + LMS). At the end of the experiments, there was no significant difference in body weight between the control group and the Cy-treated group. Interestingly, seven days after modeling, the weight gain of mice in the Cy-treated groups remarkably decreased (*p* < 0.05) compared to the control group, and weight gain in the SCP-treated group (except for Cy+MSCP) significantly increased (*p* < 0.05) compared to the Cy-treated group. However, supplementation with the original CP did not increase weight gain compared to the Cy group. The thymus and spleen are important immune organs, and their organ index can reflect immune function to a certain extent [1]. As shown in Table 1, the spleen index increased in mice treated with SCP, particularly in the low dose group that showed a significant increase (*p* < 0.05) in the spleen index compared with the Cy group. This suggests that SCP treatment mitigated spleen atrophy. In contrast, there was no significant change in the thymus index in the control group or the Cy-treated groups.

### 2.3. Effects of SCP on the Expression of IFN-γ, IL-2, IL-6, and TNF-α in Mice

**Figure 2:**
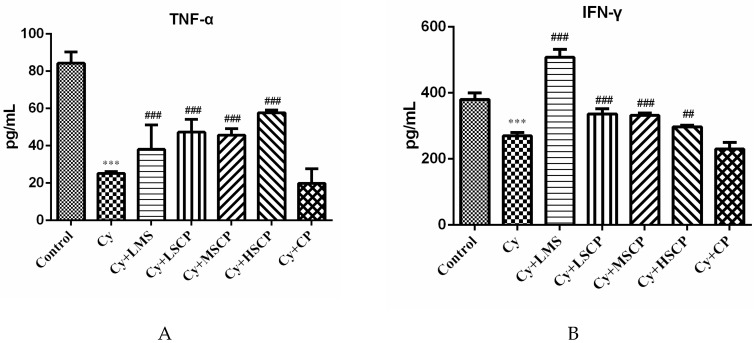
Effects of subcritical water-hydrolyzed citrus pectin (LSCP, MSCP, and HSCP at 300, 600, and 1200 mg/kg·bw, respectively) and original citrus pectin (1200 mg/kg·bw) on the levels of serum cytokines (A) TNF-α and (B) IFN-γ in mice. The values expressed as mean ± SD, n = 5. *** *p* < 0.001, compared to the control group; ## *p* < 0.01, ### *p* < 0.001, compared to the Cy group.

Cytokines act through receptors and are important to immune response and regulation [20]. Thus, the levels of cytokines in the sera of the Cy-induced ICR mice were determined. As shown in Figure 2, the secretion levels of TNF-α and IFN-γ in the Cy group were significantly decreased compared with the control group; however, Cytreatment did not affect the secretion of IL-2 or IL-6 in the serum (data not shown). In addition, the administration of SCP and levamisole significantly increased the serum concentration of cytokines. In contrast, supplementation with the original CP did not significantly increase the levels of serum cytokines in mice (Figure 2).

**Figure 3:**
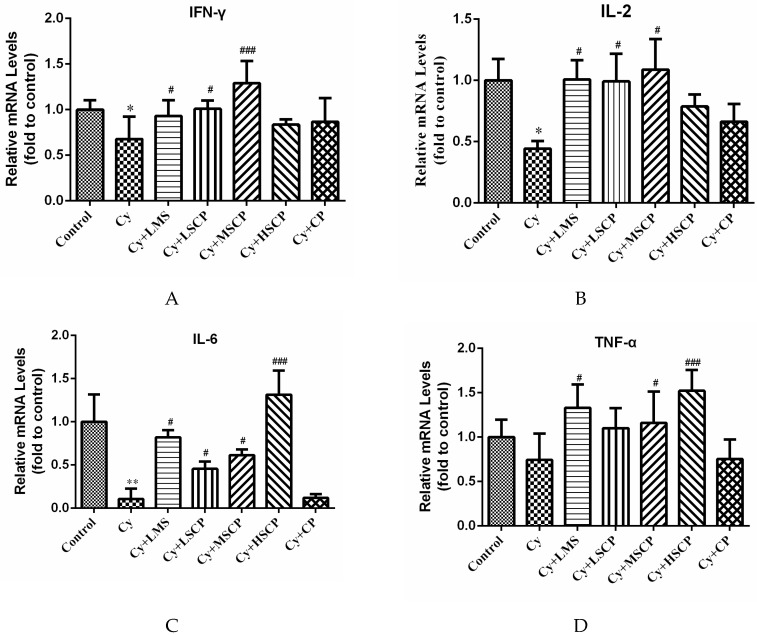
Effects of subcritical water-hydrolyzed citrus pectin (LSCP, MSCP, and HSCP at 300, 600, and 1200 mg/kg·bw, respectively) and original citrus pectin (1200 mg/kg·bw) on the relative mRNA expression of (**A**) IFN-γ, (**B**) IL-2, (**C**) IL-6, and (**D**) TNF-α in colonic tissues. The values expressed as mean ± SD, n = 5. * *p* < 0.05, ** *p* < 0.01, compared to the control group; # *p* < 0.05, ### *p* < 0.001, compared to the Cy group.

Furthermore, mRNA expression of IFN-γ, IL-2, IL-6 and TNF-α in colonic tissues was determined by qRT-PCR to investigate whether SCP regulated immunomodulatory-related genes in the Cy-induced mice. As shown in Figure 3, results showed a similar trend to that observed in serum. Compared with the control group, Cy significantly decreased mRNA expression levels of IFN-γ, IL-2, and IL-6 (*p* < 0.05) in the colon. SCP and levamisole treatment (except for the CP group) induced the up-regulation of IFN-γ, IL-2, IL-6, and TNF-α. In general, medium and high doses of SCP exhibited superior promotion of cytokine mRNA levels. Our results indicate that subcritical water-treated citrus pectin has regulatory effects on the enhancement of immunity through the secretion of these cytokines.

### 2.4. Effects of SCP on the Gut Microbiota

**Figure 4:**
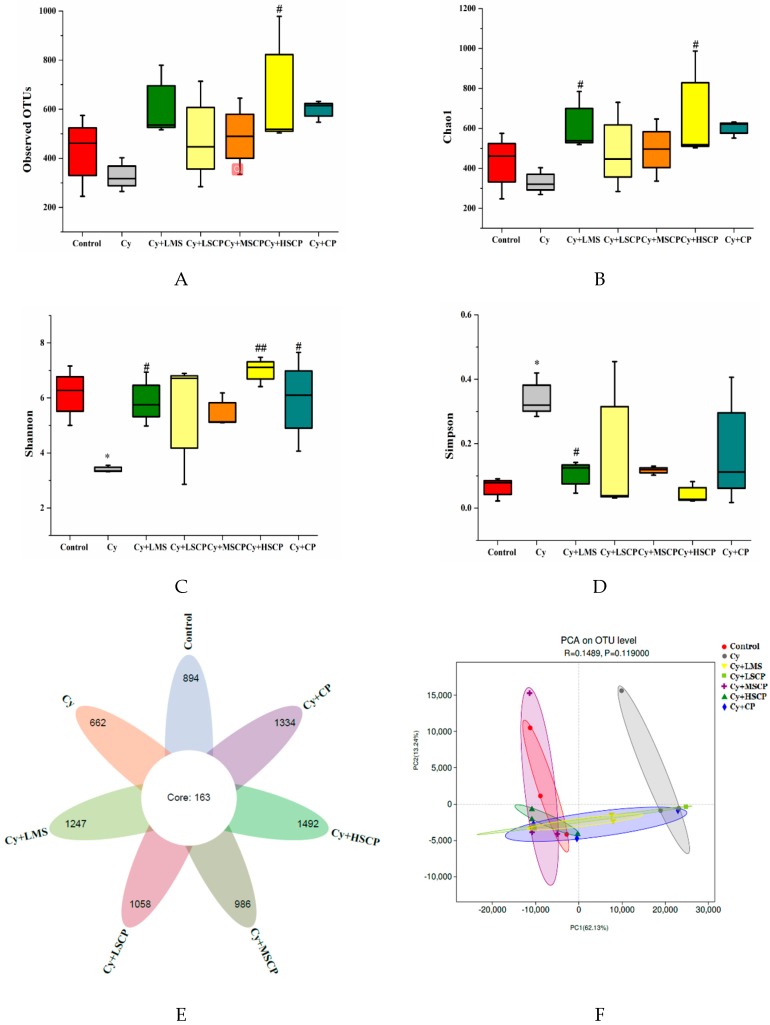
Diversity of bacterial communities and the structures of the mouse intestinal microbiota in different treatment groups. (**A–D**) Alpha diversity was evaluated by (**A**) observed OTUs, (**B**) Chao1 index, (**C**) Shannon index, and (**D**) Simpson index. (**E**) Venn diagrams of the five groups. (**F**) Principal coordinate analysis based on OTU. The values expressed as mean ± SD, n = 5. * *p* < 0.05, compared to the control group; # *p* < 0.05, ## *p* < 0.01 compared to the Cy group.

To assess the effect of SCP on fecal microbiota composition, the V3–V4 regions of the bacterial 16S ribosomal RNA genes were amplified and sequenced on an IlluminaMiSeq platform. A total of 1,684,738 sequences were obtained from 21 samples, with an average of 80,226 tags per mouse. Following chipping and filtering, the number of clean reads available for subsequent analysis was 1,530,927, with an average of 72,901 effective tags per mouse; more than 90.87% of the effective sequences could be used for further analysis. Rarefaction curves and Shannon indices suggested that the sequencing depth was deemed to be sufficient (Figure S1). As shown in Figure 4E, there were 7836 OTUs identified at the 97% similarity level, and there were 163 OTUs shared among the seven groups. There were 894, 662, 1247, 1058, 986, 1492 and 1334 OTUs identified in the control, Cy, Cy + LMS, Cy + LSCP, Cy + MSCP, Cy + HSCP and Cy + CP groups, respectively. The alpha diversity analysis, Chao 1, Simpson index, Shannon index, and observed OTUs were calculated to evaluate microbial diversity and richness. Compared with the control group, significantly decreased community diversity of fecal microbiota was observed in the Cy group based on the Simpson and Shannon indices (*p* < 0.05, Figure 4 C and D). Compared with the Cy-treated group, Chao 1, Shannon index, and observed OTUs were increased in the SCP treated groups. In addition, the Simpson index was significantly decreased in these groups, revealing that the SCP treatments increased microbial diversity and richness [17]. The differences in gut microbiota between treatments were analyzed via the beta diversity metric using principal component analysis (PCA). As shown in Figure 4F, the Cy-treated mice exhibited obvious divergences in the community structure of the intestinal flora compared to the control mice. However, the SCP treatments altered the microbiota so that it was similar to that of the control group. Likewise, levamisole and the original CP intervention modulated gut microbiota such that it was different from the Cy-treated group, while the Cy + LMS and Cy + CP groups clustered further from the control mice than the SCP-treatment mice. This suggests that SCP reverted the gut microbiota composition in the cy-induced immunosuppressed mice to a more normal status.

**Figure 5:**
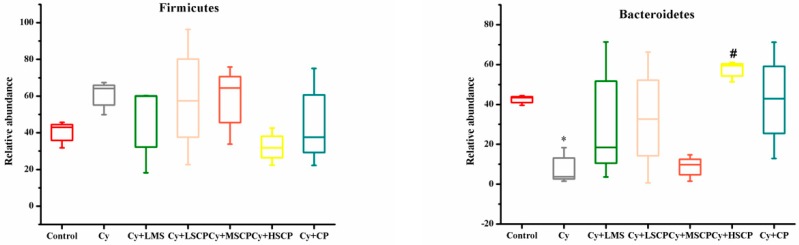

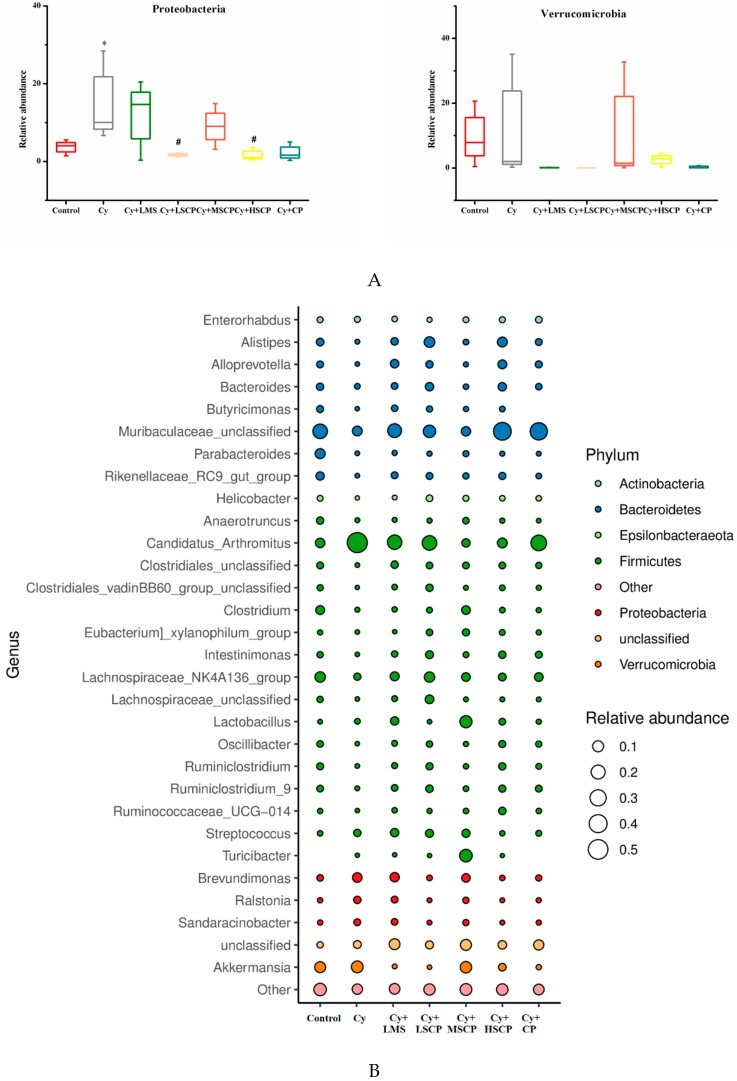
SCP supplementation altered the composition of gut microbiota in cyclophosphamide-induced immunosuppressed mice. (**A**) The relative abundances of Firmicutes, Bacteroidetes, Proteobacteria and Verrucomicrobia. (**B**) Bubble plot of gut microbiota composition. * *p* < 0.05, ** *p* < 0.01, *** *p* < 0.001, compared to the control group; # *p* < 0.05, ## *p* < 0.01, ### *p* < 0.001, compared to the Cy group.

The major phyla in the experimental mice included Proteobacteria, Bacteroidetes, and Firmicutes. At the phylum level, the relative abundance of Bacteroides was remarkably reduced in the Cy-treated group (42.43 ± 2.56 in the control group vs. 7.87 ± 5.13 in the Cy group, *p* < 0.05). The relative abundance of Firmicutes increased (40.16 ± 7.41 in the control group vs. 60.47 ± 9.34 in the Cy group) when compared to control group (Figure 5 and Figure S2), which is consistent with the major reduction in the Bacteroidetes and Firmicutes ratio observed in the cy-induced immunosuppressed mice [3]. As shown in Figure 5 and Figure S2, SCP normalized the abundance of Bacteroidetes and Firmicutes (57.27 ± 5.26 for Bacteroidetes and 32.25 ± 10.14 for Firmicutes in the Cy + HSCP group). Moreover, SCP supplementation fully prevented an increase in the abundance of Proteobacteria, the largest of the bacterial domains that include many pathogenic bacteria. At the family level, of the main 15 families, Muribaculaceae, Lachnospiraceae, Ruminococcaceae, Rikenellaceae, Prevotellaceae, and Bacteroidaceae were less abundant, while Clostridiaceae, Akkermansiaceae, Streptococcaceae, Caulobacteraceae, Sphingomonadaceae, and Burkholderiaceae were more abundant in the Cy than in the control group (Figure S3). However, following supplementation with SCP, the relative abundance of these bacteria reverted to levels like that of the ND mice (*p* > 0.05; Figure S3). A bubble plot was created to demonstrate the differences between groups at the genus level (Figure 5B). Cy-treated mice showed remarkably elevated levels of *Brevundimonas*, *Streptococcus* and *Candidatus_Arthromitus*, while the abundance of *Muribaculaceae_unclassified*, *Lachnospiraceae_NK4A136_group*, *Bacteroides*, *Clostridium*, *Rikenellaceae_RC9_gut_group*, *Ruminiclostridium_9*, *Lachnospiraceae_unclassified*, and *Helicobacter* was reduced. Again, the relative abundance of the above genera was restored in the SCP-treated mice. These results implied that SCP administration can modulate the community structure of the intestinal flora in immunosuppressed mice, resulting in a microbiota composition like that of control mice.

**Figure 6:**
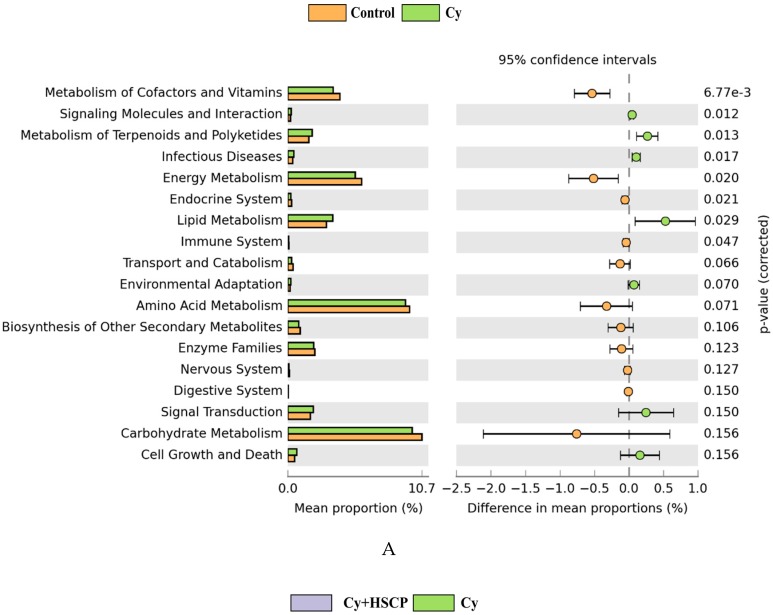

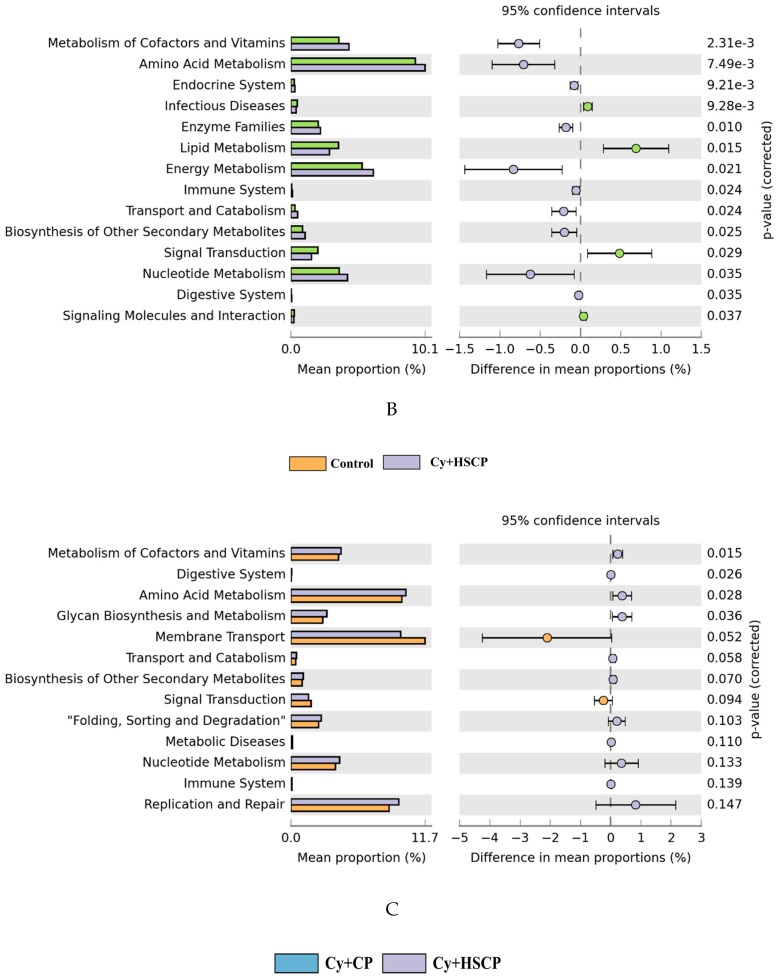

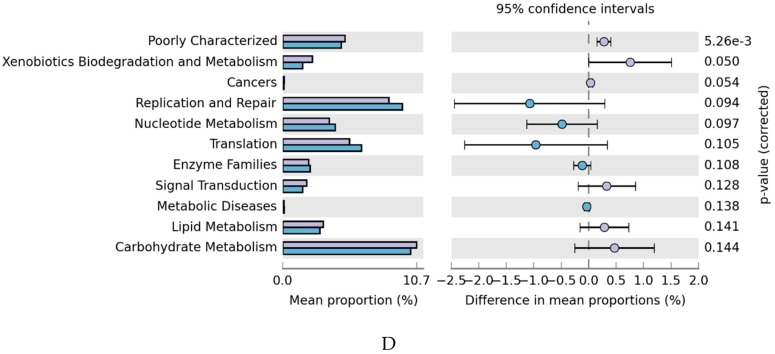
Functional prediction of altered gut microbiota based on the KEGG pathways. (**A**) Control group *vs* Cy group; (**B**) Cy + HSCP group *vs* Cy group; (**C**) Control group *vs* Cy + HSCP group; (**D**) Cy + CP group *vs* Cy + HSCP group.

In addition to taxonomic composition, a PICRUSt tool was used to predict the metabolic function of the microbiota induced by Cy. It was determined that the metabolism of cofactors and vitamins, energy metabolism, immune system, and lipid metabolism were significantly altered in Cy-treated mice (Figure 6A). Notably, supplementation with SCP in immunosuppressed mice contributed to the functional difference in microbiota at KEGG levels, similar to the control group. As shown in Figure 6B, SCP-induced functional differences in microbial communities, including 10 enriched (metabolism of cofactors and vitamins, amino acid metabolism, energy metabolism, immune system, transport, and catabolism) and four depleted (lipid metabolism, signal transduction, signaling molecules and interaction, and infectious diseases) compared with the Cy group. Collectively, these results indicated that treatment with SCP balanced the microbial structure, composition, and metabolism of the gut microbiota to sustain immunity.

## 3. Discussion

In the present study, subcritical water-hydrolyzed citrus pectin exhibited improved functional properties and bioactivities on immunoregulation, including increased weight gain and spleen index, and contributed to the enhanced expression of IFN-γ, IL-2, IL-6, and TNF-α, compared to the original citrus pectin. Furthermore, the structure and relative abundance of the gut microbiota was changed in Cy-induced immunosuppressed mice. Emerging evidence shows that degradation or modification of pectin can increase its biological activities, mainly due to the generation of smaller fragments with lower molecular mass and increased solubility, making it possible to enter the digestive system [12,13]. In this study, we showed that subcritical water treatment had no effect on the primary structure of pectin, according to the results of FT-IR analysis (Figure 1). However, subcritical water treatment effectively decreased the molecular mass of citrus pectin from 157 kDa to 123 kDa for Mw and 121 kDa to 86 kDa for Mn. Furthermore, the galacturonic acid content was increased after subcritical water hydrolysis. Previous studies showed that the application of ultrasound pretreatment gives rise to a higher content of galacturonic acid and a lower molecular mass, therefore improving its biological activities such as anti-cancer and antioxidant activity [12,13]. These findings proved that lower a molecular mass and altered properties of pectin might contribute to the immunoregulatory effects of SCP in Cy-induced immunosuppressed mice.

Cy is an important alkylating agent used in cancer treatment and blood and marrow transplantation [21]. However, the administration of Cy can lead to immunosuppression and hepatotoxicity that is reflected in lower body weight and spleen and thymus indices, disruption of the intestinal mucosal immunity, liver dysfunction, and oxidative stress [22,23]. Thus, Cy was used to creating the immunosuppressed animal model in this study. Cy-induced immunosuppressed mice exhibited significantly lower body weight gain, spleen index, and cytokine levels (IFN-γ, IL-2, IL-6, and TNF-α) compared to the control group [24]. Similar changes were also shown in the present Cy-induced mouse model, indicating that the immunosuppression model was built successfully. The spleen is an important immune organ in nonspecific immune regulation, where the T and B cells colonize, synthesize biologically active substances, and produce an immune response [25]. Cy injection caused a decrease in the spleen index, suggesting that the immune function of spleen decreased, and the spleen itself shrank. However, mice treated with SCP had a significantly increased spleen index (especially in the medium-dose group; *p* < 0.05; Table 1), indicating that SCP can weaken Cy-induced immunosuppressive activity. Additionally, the shrinking of the spleen and decreased immune function can produce innate immune responses via the regulation of cytokine levels. Cytokines are a series of functional immune regulators synthesized and secreted by immune cells (T cells, B cells, and NK cells) and non-immune cells (epidermal cells, endothelial cells and fibroblasts). Cytokines are involved in the preservation or restoration of homeostasis via coordination of lymphoid cells, inflammatory cells, and hematopoietic cells [26]. Different cytokines often play different roles in the host defense system. For example, IL-2, IL-12, IFN-γ, and TNF-α secreted by Th1 cells promote cell-mediated immune responses, while IL-4, IL-5, IL-6, and IL-10 produced by Th2 cells are necessary for humoral or allergic responses [27]. Our results indicated that supplementation with SCP remarkably (*p* < 0.05) promoted serum TNF-α and IFN-γ secretion, and the mRNA expression of IFN-γ, IL-2, IL-6, and TNF-α in colonic tissues was also significantly increased. These results are in agreement with previous studies showing that polysaccharides from plants can enhance the immune organ index and the production of cytokines [6,7,17]. 

Immune system dysregulation is often accompanied by the dysbiosis of intestinal microbiota. According to Xu et al. [3], Cy-induced immunosuppression is closely related to dysbiosis of the intestinal microbiota, stimulating the proliferation of Proteobacteria and Firmicutes and decreasing the proportion of Bacteroidetes. Furthermore, various polysaccharides can modulate the composition of the gut microbiota altered by Cy, thereby regulating host immunity [5,7]. Thus, the characteristics of the gut microbiota in the Cy-induced immunosuppressed mice with or without SCP, and the original CP intervention was investigated. Our results showed that treatment with SCP prevented adecrease in microbial richness and diversity in the Cy-induced immunosuppressed mice (Figure 4A–D). We further observed that SCP supplemented mice were similar in their microbiota profiles to the control mice. However, although the original CP intervention modulated gut microbiota to distinguish it from that of the Cy-treated group, it also clustered far from that of the control mice. SCP, rather than the original CP, reverted the composition of gut microbiota in the immunosuppressed mice to normal status. This may due to the lower molecular mass, changed the molecular structure, and physical characteristics of SCP, improving its metabolism by the gut microbiota [28]. Our work agrees with previous reports showing that Firmicutes/Bacteroidetes ratio, as well as immunosuppression, could be ameliorated in the Cy-induced mice receiving supplementation with pectic heteropolysaccharide [7]. Moreover, the abundance of Proteobacteria, which are related to intestinal diseases [1], was significantly increased by Cy treatment, while the relative abundance of Proteobacteria was decreased by SCP treatment. Furthermore, it was also proved that the decreased abundance of the dominant family Muribaculaceae, which belongs to the Bacteroidetes family, was significantly increased by SCP treatment in the Cy-induced mice (Figure S4). The Muribaculaceae family (previously called S24-7) commonly dominates the mouse fecal microbiota. Immune-driven dysbiosis with a disproportion of Muribaculaceae was also observed in immune-deficient mice [3]. Specifically, the present study showed that the SCP-treated mice had an increased abundance of bacteria that have been previously associated with the secretion of inflammatory cytokines, including Muribaculaceae_unclassified and Bacteroides [29]. On the contrary, the potentially pathogenic bacteria Brevundimonas, which belong to the Proteobacteria and is related to intestinal diseases [30], was inhibited by SCP treatment. SCP not only inhibited the increase of pathogenic bacteria but also promoted the growth of beneficial bacteria, including Lachnospiraceae, Ruminococcaceae, Rikenellaceae, Prevotellaceae and Bacteroidaceae. Lachnospiraceae can hydrolyze various plant polysaccharides and promote intestinal health [31]. Ruminococcaceae, Bacteroidaceae and Prevotellaceae were reported to be positively associated with the production of SCFAs [32], which can promote the production of colonic regulatory T cells induced by histone H3 acetylation [33]. Additionally, PICRUSt analysis showed enrichment in KEGG pathways of the immune system in the SCP group, which may be associated with the immune response. These changes might be a crucial mechanism for the immunoregulatory effects of SCP. Further, metagenomics studies are required to verify the effects of SCP on certain metabolic pathways. Collectively, and consistent with the previous research, we conclude that SCP showed a prebiotic effect on gut microbiota in the cy-treated mice, which may be responsible for the amelioration of immunosuppression.

## 4. Materials and Methods

### 4.1. Preparation of Subcritical Water-Hydrolyzed Citrus Pectin

Citrus pectin was obtained from Yantai Andre Pectin Co., Ltd. (Yantai, China). Citrus pectin powder was dissolved in distilled water for 30 min at 45 °C and then passed through a filter paper to obtain the final pectin concentration of 1.5% (w/v). The pectin solution was hydrolyzed in subcritical water at 120 °C for 40 min, as described in [34], using a D-1 high-pressure pump (Beijing Fa’en Technology and Trade Co., Ltd., Beijing, China). The degraded pectin solution was collected and freeze-dried (SCIENTZ-18N; Ningbo Scientz Biotechnology Co., LTD, Ningbo, China). The dried pectin was stored at −18°C until use.

### 4.2. The Properties of Subcritical Water-Hydrolyzed Citrus Pectin

The molecular weight of subcritical-water hydrolyzed citrus pectin (SCP), and the original citrus pectin (CP) was measured by size exclusion chromatography using an ultrahydrogel linear column (7.8 × 300 mm, Waters, Milford, MA, USA) as described in Klinchongkon et al. [16]. The galacturonic acid content in SCP and CP was determined by the carbazole method using an ultraviolet spectrophotometer (UV-2600, Unico Instruments Co., Ltd., Shanghai, China) at 530 nm [35]. The dried citrus pectin sample was mixed with potassium bromide (KBr) powder, and the Fourier-transform infrared spectra (FT-IR) was obtained on a Nicolet iS10 FT-IR spectrometer (Thermo Fisher Scientific, Waltham, MA, USA) in the range of 4000–400 cm^−1^.

### 4.3. Animals and Experimental Design

Thirty-five 6-week-old male ICR mice were purchased from Shanghai SLAC Laboratory Animal Company Limited (Shanghai, China). Mice were cared for in accordance with the National Institutes of Health Guide for Care and Use of Laboratory Animals (Publication No. 85-23, revised 1996), and all experimental procedures were approved by the Animal Care Review Committee, Hangzhou Normal University, Hangzhou, China (Protocol No. 2019168). Animals were group-housed (five mice per cage) at 21 ± 2°C and 60 ± 5% relative humidity room under a 12-h light/dark cycle. All animals have free access to food and water.

After a one-week acclimation, the animals were distributed into seven groups (n = 5): control group, cyclophosphamide group (Cy), positive group treated with levamisole (Cy + LMS), three concentrations of SCP (Cy + LSCP: 300 mg/kg, Cy + MSCP: 600 mg/kg, Cy + HSCP: 1200 mg/kg) and the original CP group (Cy + CP: 1200 mg/kg). The SCP and CP groups were administered SCP (300, 600, or 1200 mg/kg) and CP (1200 mg/kg) once daily for 21 continuous days by intragastric gavage. The levamisole group was injected subcutaneously with levamisole at a dose of 7 mg/kg bw for 21 continuous days; the control and Cy groups received an equal volume of saline via gavage. In addition to the control group, mice in the other groups were injected subcutaneously with cyclophosphamide (40 mg/kg/d) at days 13, 14, and 16 to induce immunosuppression. Bodyweight was monitored twice per week. All reagents were dissolved aseptically in physiological saline.

### 4.4. Analysis of Immune Organ Indices

All mice were weighed before sacrifice. Then, the mice were sacrificed by CO2 inhalation after being anesthetized with ether. The thymus and spleen of each mouse were surgically excised and weighed, and the thymus and spleen indices were calculated using the following formula:

Thymus index = Thymus weight (mg)/body weight (g)(1)

Spleen index = Spleen weight (mg)/body weight (g)(2)

### 4.5. Quantification of Cytokines by ELISA

The content of serum cytokines was measured by ELISA. Blood samples were obtained from the mice via eyeball enucleation following sacrifice, and then serum was collected by centrifugation (4 °C, 1200 g, 20 min). Cytokine levels (IFN-γ, IL-2, IL-6, and TNF-α) were detected via the mouse ELISA kit (Wuhan Huamei Biotechnology Co., Ltd., Hunan, China), according to the manufacturer’s instructions.

### 4.6. Quantitative Real-Time PCR

Total RNA isolated from colon tissue was used for cDNA synthesis using the methods previously described [36]. Gene expression was assessed by the 2−ΔΔCt method, and gene expression levels were normalized to the housekeeping gene, Glyceraldehyde-3-phosphate dehydrogenase (GAPDH). Primer sequences (IFN-γ, IL-2, IL-6, TNF-α, and GAPDH) are available in Table S1.

### 4.7. Fecal DNA Extraction and Pyrosequencing

Fecal samples were freshly collected at the end of day 21 and immediately stored at −80 °C. Bacterial genomic DNA was extracted using a DNA Extraction Kit (Omega Bio-Tek, Norcross, GA, USA). Each DNA sample was subsequently used for 16S amplification of the V3-V4 region using the primers 341F (5’-CCTACGGGNGGCWGCAG-3’) and 805R (5’-GACTACHVGGGTATCTAATC-C-3’). The gut microbiota composition was analyzed on an IlluminaMiSeq platform, as described previously [37]. The bioinformatic analysis is provided in the Supporting Information section.

### 4.8. Statistical Analysis

Statistical analysis was performed using GraphPad Prism 6.0 software (La Jolla, CA, USA) and SPSS 20.0 software (Chicago, IL, USA). Data were presented as mean ± SD (n = 5) or box-whisker plots. Statistical differences between two groups were analyzed by one-way ANOVA followed by an LSD post hoc test, and *p* < 0.05 was considered statistically significant. Statistical analysis and visualization of the Kyoto Encyclopedia of Gene and Genomes (KEGG) pathway (Level 2) were performed using STAMP 2.1.3 software by Welsh’s t-test.

## 5. Conclusions

In this work, SCP exhibited improved functional properties and bioactivities on immunoregulatory effects compared to the original citrus pectin, which might due to the smaller fragments with a lower molecular mass and increased galacturonic acid content. Concomitantly, dietary SCP increased body weight gain and the spleen index and contributed to enhanced expression of IL-2, IL-6, IFN-γ, and TNF-α in the Cy-induced immunosuppressed mice, resulting in improved immune function. Furthermore, SCP also ameliorated the gut microbiota composition in the Cy treated mice, as an increased abundance of beneficial bacteria including Muribaculaceae, Ruminococcaceae, Bacteroidaceae, and Prevotellaceae, and a decreased abundance of pathogenic Brevundimonas and Streptococcus was observed. We thus suggest that SCP is a potential immunomodulator and may be important in the prevention and treatment of immunosuppressive diseases.

## Figures and Tables

**Table 1:** Effects of subcritical water-hydrolyzed citrus pectin (LSCP, MSCP, and HSCP at 300, 600, and 1200 mg/kg·bw, respectively) and original citrus pectin (1200 mg/kg·bw) on body weight and immune organ indices in immunosuppressed mice.

Groups	Body Weight (g)	Body Weight Gain after Treatment with Cy (g)	Thymus Index (mg/g)	Spleen Index (mg/g)
Control	33.38±1.32	5.24 ± 1.57	1.47 ± 0.38	2.72 ± 0.67
Cy	31.62 ± 1.94	3.61 ± 0.54 ^*^	1.39 ± 0.01	2.31 ± 0.50
Cy + LMS	32.46 ± 1.16	4.62 ± 0.72 ^#^	1.26 ± 0.26	2.70 ± 0.24
Cy + LSCP	31.76 ± 2.93	4.22 ± 1.34 ^#^	1.31 ± 0.24	3.06 ± 0.26 ^#^
Cy + MSCP	30.02 ± 2.29 ^*^	3.75 ± 0.90	1.40 ± 0.41	2.65 ± 0.29
Cy + HSCP	30.72 ± 2.63 ^*^	4.14 ± 0.83	1.18 ± 0.24	2.41 ± 0.33
Cy + CP	29.50 ± 1.83 ^***^	3.02 ± 1.16 ^*^	1.20 ± 0.25	2.46 ± 0.34

LSCP, low-dose SCP; MSCP, middle-dose SCP; HSCP, high-dose SCP. The values expressed as mean ± SD, n = 5. * *p* < 0.05, ** *p* < 0.01, *** *p* < 0.001, compared to the control group; # *p* < 0.05, ## *p* < 0.01, ### *p* < 0.001, compared to the Cy group.

## Supplementary Materials

The following are available online: Table S1: PCR primers used in the present study. Table S2. The abundance of intestinal flora atrhw genus level. Figure S1: Effects of subcritical water-hydrolyzed citrus pectin (LSCP, MSCP, and HSCP at 300, 600 and 1200 mg/kg bw, respectively) and original citrus pectin (1200 mg/kg bw) on the levels of serum cytokines (A) IL-2 and (B) IL-6 in mice. Figure S2: Rarefaction analysis (A) and Shannon index (B) of all samples. Figure S3: The abundance of intestinal flora at the family level of the top 15.
