# Supplementary material for: Treatment with Subcritical Water-Hydrolyzed Citrus Pectin Ameliorated Cyclophosphamide-Induced Immunosuppression and Modulated Gut Microbiota Composition in ICR Mice
Source: Molecules. 2020 Mar 12;25(6):1302. doi: 10.3390/molecules25061302 (PMC7144127; doi:10.3390/molecules25061302)
Supplement: Supplementary file 1 [file molecules-25-01302-s001.pdf]

Treatment with Subcritical Water-Hydrolyzed Citrus Pectin Ameliorated Cyclophosphamide-Induced Immunosuppression and Modulated Gut Microbiota Composition in ICR Mice

Authors: Jianbing Chen[1,2], Chengcheng Zhang[2], Qile Xia[2], Daqun Liu[2], Xinghe Tan[1,*], Yingdi Li[2] and Yan Cao[2]

[1] College of Food Science and Technology, Hunan Agricultural University

[2] Food Science Institute, Zhejiang Academy of Agricultural Sciences, Key Laboratory of Post-Harvest Handling of Fruits, Ministry of Agriculture and Rural Affairs, Hangzhou 310021, China

*Correspondence: Xinghe Tan, xinghetan@163.com

Fecal microbiota analysis

Generated and demultiplexed sequences were analyzed using the QIIME software package (version 1.17). Resulting sequences containing ambiguous or low quality reads (Phred score ≤ 25) were removed from the dataset; reads with at least one reverse primer mismatch or where the reverse primer was not found were discarded. Sequences that overlapped longer than 10 bp based on their overlap sequence were merged, while those reads that could not be assembled were discarded using FLASH [1] For each faecal sample, the remaining unique reads were clustered into operational taxonomic units by UPARSE based on RDP with 97% kinsmanship cutoff. Representative OTU sequences were assigned taxonomy against the Greengenes reference database (August 2013 release) using the RDP-classifier. Mothur was used to evaluate alpha-diversities including Observed, Chao, Ace, Shannon, Simpson and Coverage indexes, as well as Rarefraction, Shannon curves. R package (V3.4.0) was applied to estimate beta-diversities including Principal coordinate analysis (PCoA) [2]. To predict the functional profiles of microbial communities of cyclophosphamide-induced immunosuppressed mice, phylogenetic investigation of communities by reconstruction of unobserved states (PICRUSt) analysis was performed. The functions were estimated based on 16S rRNA sequencing data and the Kyoto Encyclopedia of Genes and

Genomes (KEGG) database [3].

References

1 Magoc, T.; Salzberg, S.L. FLASH: fast length adjustment of short reads to improve genome assemblies. Bioinformatics. 2011, 27, 2957-2963.

2 Schloss, P.D.; Gevers, D.; Westcott, S.L. Reducing the effects of pcr amplification and sequencing artifacts on 16S rRNA-based studies. Plos One. 2011, 6.

3 Langille, M.G.I.; Zaneveld, J.; Caporaso, J.G.; McDonald, D.; Knights, D.; Reyes, J.A.; Clemente, J.C.; Burkepile, D.E.; Thurber, R.L.V.; Knight, R.; Beiko, R.G.; Huttenhower, C. Predictive functional profiling of microbial communities using 16S rRNA marker gene sequences. Nat BioTechnol. 2013, 31, 814.

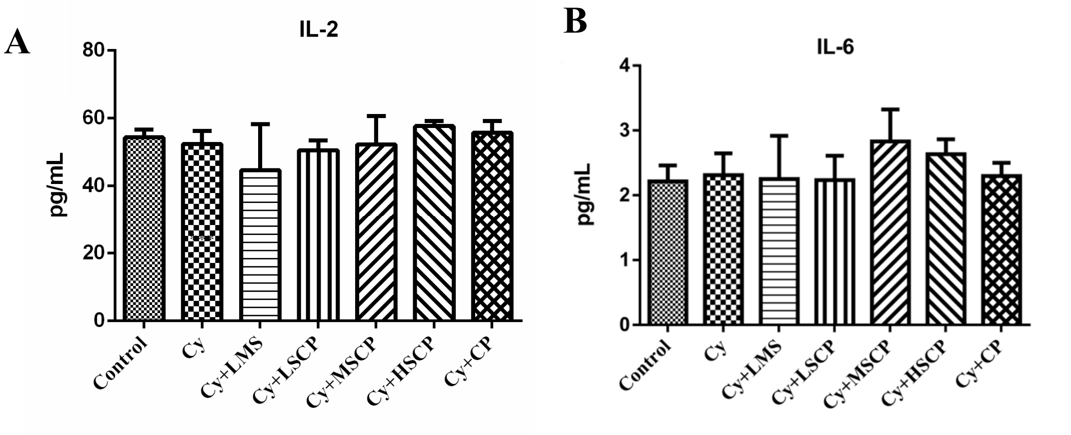

Figure S1. Effects of subcritical water-hydrolyzed citrus pectin (LSCP, MSCP and HSCP at 300, 600 and 1200 mg/kg·bw, respectively) and original citrus pectin (1200 mg/kg·bw) on the levels of serum cytokines (A) IL-2 and (B) IL-6 in mice.

A

B

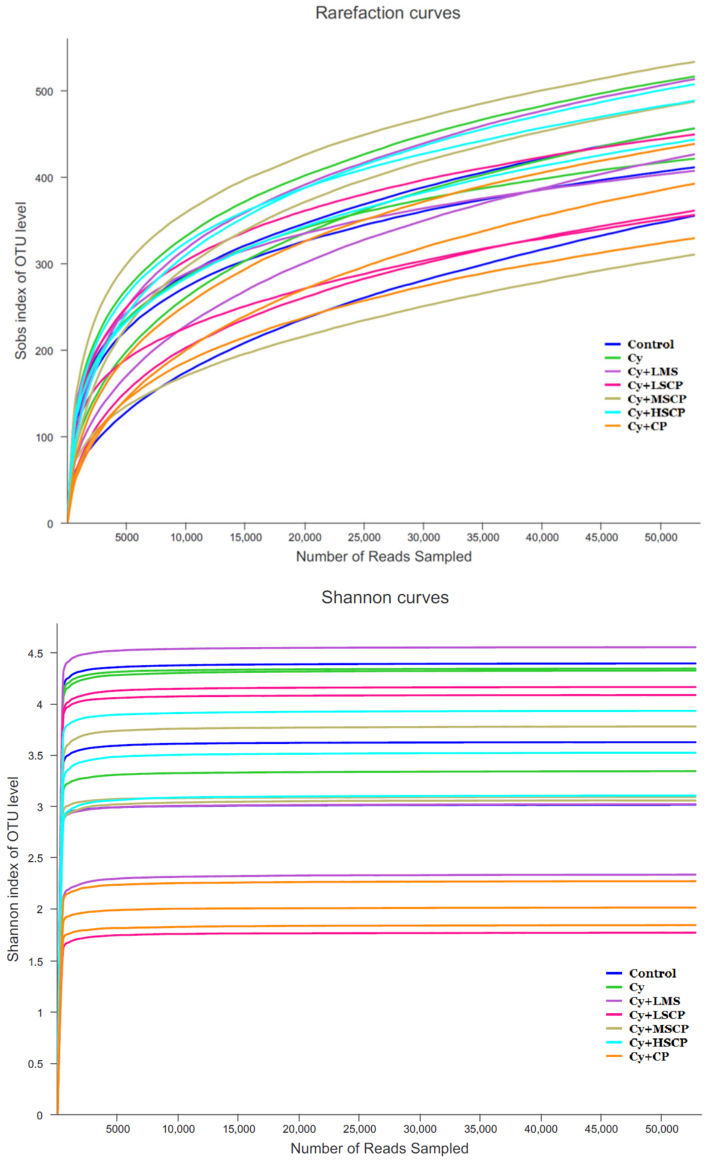

Figure S2. Rarefaction analysis (A) and Shannon index (B) of all samples. Each bar represents one sample.

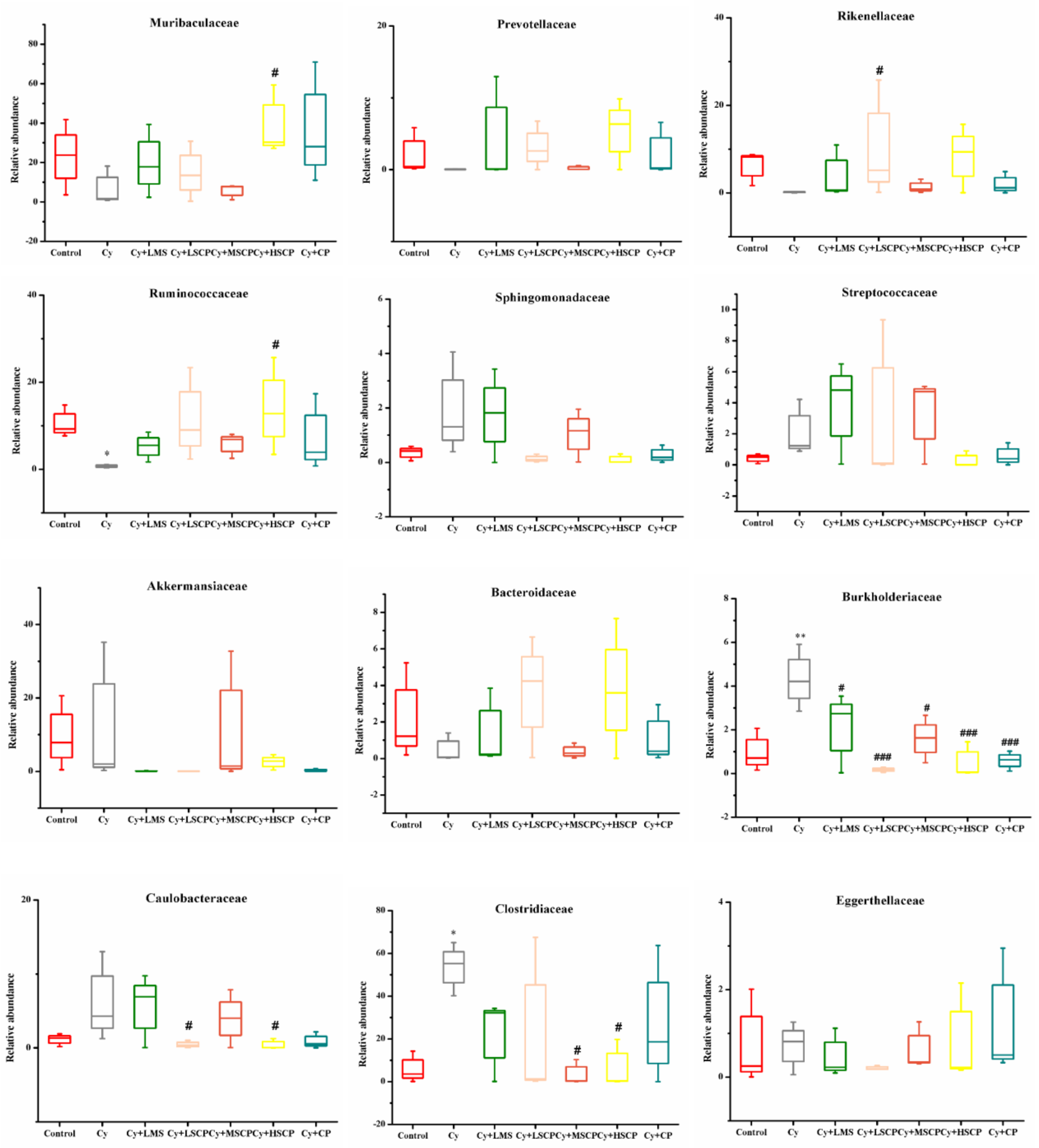
Muribaculaceae
Prevotellaceae
Rikenellaceae
Ruminococcaceae
Sphingomonadaceae
Streptococcaceae
Akkermansiaceae
Bacteroidaceae
Burkholderiaceae
Caulobacteraceae
Clostridiaceae
Eggerthellaceae
Relative abundance
Control
Cy
Cy+LMS
Cy+LSCP
Cy+MSCP
Cy+HSCP
Cy+CP
#
*
**
###

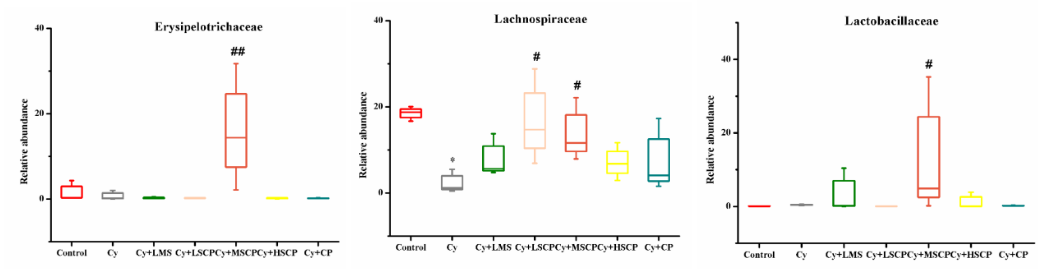

Figure S3. The abundance of intestinal flora at family level of top 15.

*p < 0.05, ** p < 0.01, *** p < 0.001, compared to Control group;
# p < 0.05, ## p < 0.01, ### p < 0.001, compared to Cy group.

Table S1. PCR primers used in the present study.

Name	Sequence（5'-3')
IL6-F	TACCACTTCACAAGTCGGAGGC
IL6-R	CTGCAAGTGCATCATCGTTGTTC
IL2-F	GCGGCATGTTCTGGATTTGACTC
IL2-R	CCACCACAGTTGCTGACTCATC
IFN-F	CAGCAACAGCAAGGCGAAAAAGG
IFN-R	TTTCCGCTTCCTGAGGCTGGAT
TNF-F	GGTGCCTATGTCTCAGCCTCTT
TNF-R	GCCATAGAACTGATGAGAGGGAG
GAPDH,F	TGCACCACCAACTGCTTAGC
GAPDH,R	GGCATGGACTGTGGTCATGAG

Table S2. The abundance of intestinal flora at genus level

Genus level	Control	Cy+ HSCP	Cy+ CP	Cy+ LSCP	Cy+ MSCP	Cy+ LMS	Cy
Muribaculaceae_unclassified	22.66	38.44	35.88	14.53	5.61	19.70	6.89
Candidatus_Arthromitus	5.97	6.74	27.47	23.02	3.61	22.08	53.38
unclassified	0.98	3.91	7.75	3.11	9.83	9.50	2.79
Akkermansia	9.64	2.56	0.27	0.03	11.42	-	12.46
Lachnospiraceae_NK4A136_group	8.19	2.82	4.18	7.95	4.31	4.81	1.88
Alistipes	2.51	6.55	1.48	8.83	0.51	2.45	-
Lactobacillus	-	1.32	0.23	-	13.43	3.55	0.45
Brevundimonas	1.14	0.45	0.91	0.47	3.96	5.56	6.17
Turicibacter	-	-	-	-	15.30	-	-
Alloprevotella	1.57	4.42	1.67	2.18	0.17	3.91	-
Streptococcus	0.43	0.31	0.61	3.15	3.28	3.79	2.11
Bacteroides	2.22	3.76	1.13	3.65	0.39	1.41	0.50
Clostridium	4.41	0.48	0.27	0.62	4.23	0.31	0.17
Rikenellaceae_RC9_gut_group	3.71	1.83	0.55	1.53	0.87	1.52	-
Intestinimonas	1.06	2.11	1.62	2.73	0.56	0.95	-
Ruminiclostridium_9	1.27	1.88	1.52	2.45	0.47	1.27	0.12
Parabacteroides	7.00	0.18	0.13	0.24	0.73	0.21	-
Clostridiales_unclassified	1.51	1.32	0.86	1.36	1.35	2.07	-
Ruminiclostridium	1.68	2.20	0.63	1.89	0.29	0.73	-
Lachnospiraceae_unclassified	1.09	0.11	0.13	4.22	0.10	0.60	-
Oscillibacter	1.14	1.82	0.92	1.09	0.21	0.65	-
Ralstonia	0.33	0.11	0.25	0.12	1.10	1.61	2.18
Enterorhabdus	0.76	0.70	1.26	0.21	0.64	0.46	0.68
Sandaracinobacter	0.30	-	0.22	0.12	0.87	1.42	1.60
Butyricimonas	1.80	0.46	0.00	0.87	0.20	1.11	0.02
Clostridiales_vadinBB60_group_unclassified	0.80	0.55	0.22	2.04	0.13	0.52	-
Anaerotruncus	2.11	0.26	-	0.22	1.23	0.14	-
Eubacterium]_xylanophilum_group	0.25	0.65	0.24	1.10	1.82	-	-
Ruminococcaceae_UCG-014	0.33	2.27	0.36	0.21	0.38	0.41	-
Helicobacter	0.64	0.37	0.44	1.41	0.78	-	-
Others	14.45	11.30	8.69	10.61	12.21	9.06	7.89

Bacteria with abundance higher than 0.1% were listed.

"-" indicates that the abundance was lower than 0.1% or had not been tested.
